# Neurodevelopmental screening in children with early-onset spinal muscular atrophy in the treatment era: a strengths-based cohort study

**DOI:** 10.1093/braincomms/fcaf272

**Published:** 2025-07-21

**Authors:** Lakshmi Balaji, Didu Kariyawasam, Karen Herbert, Hugo A Sampaio, Anita Cairns, Brittany C McGill, Lauren Kelada, Susan Woolfenden, Nancy Briggs, Michelle A Farrar

**Affiliations:** Department of Neurology, Sydney Children's Hospital Randwick, Sydney, NSW 2031, Australia; Discipline of Paediatrics and Child Health, School of Clinical Medicine, UNSW Medicine and Health, University of New South Wales, Sydney, NSW 2031, Australia; Department of Neurology, Sydney Children's Hospital Randwick, Sydney, NSW 2031, Australia; Discipline of Paediatrics and Child Health, School of Clinical Medicine, UNSW Medicine and Health, University of New South Wales, Sydney, NSW 2031, Australia; Department of Neurology, Sydney Children's Hospital Randwick, Sydney, NSW 2031, Australia; Department of Physiotherapy, Sydney Children's Hospital Randwick, Sydney, NSW 2031, Australia; Department of Neurology, Sydney Children's Hospital Randwick, Sydney, NSW 2031, Australia; Discipline of Paediatrics and Child Health, School of Clinical Medicine, UNSW Medicine and Health, University of New South Wales, Sydney, NSW 2031, Australia; Queensland Children’s Hospital, Brisbane, QLD 4101, Australia; Discipline of Paediatrics and Child Health, School of Clinical Medicine, UNSW Medicine and Health, University of New South Wales, Sydney, NSW 2031, Australia; Behavioural Sciences Unit, Kids Cancer Centre, Sydney Children’s Hospital Randwick, Sydney, NSW 2031, Australia; Discipline of Paediatrics and Child Health, School of Clinical Medicine, UNSW Medicine and Health, University of New South Wales, Sydney, NSW 2031, Australia; Behavioural Sciences Unit, Kids Cancer Centre, Sydney Children’s Hospital Randwick, Sydney, NSW 2031, Australia; Central Clinical School, Sydney Medical School, the Faculty of Medicine and Health, University of Sydney, Sydney 2006, Australia; Stats Central, Mark Wainwright Analytical Centre, University of New South Wales, Sydney, NSW 2031, Australia; Department of Neurology, Sydney Children's Hospital Randwick, Sydney, NSW 2031, Australia; Discipline of Paediatrics and Child Health, School of Clinical Medicine, UNSW Medicine and Health, University of New South Wales, Sydney, NSW 2031, Australia

**Keywords:** spinal muscular atrophy, neurodevelopment, autism spectrum disorder, ages and stages questionnaire, SMN-associated neurodevelopmental disorders

## Abstract

With transformative advances in diagnostic and therapeutic approaches in spinal muscular atrophy, the long-term neurodevelopmental outcomes of children with, or predicted to have, spinal muscular atrophy type 1 are essential to evaluate. In this single-centre cross-sectional study, development in children with/at-risk of spinal muscular atrophy type 1, aged 1–66 months, was assessed using parent-reported Ages and Stages Questionnaires® (ASQ-3™). Risk of autism spectrum disorder (ASD), parental distress, sociodemographic and clinical characteristics were also evaluated. Associations between exploratory variables and developmental risk were analysed within a bioecological model of health. Adaptive least absolute shrinkage and selection operator (LASSO) was used to identify variables most predictive of developmental acquisition. Thirty-seven children with spinal muscular atrophy participated (response rate: 90.2%, girls: 54.0%). Clinical characteristics varied with modality of diagnosis, survival motor neuron 2 (*SMN2*) copies and clinical status at initiation of survival motor neuron (SMN)-augmenting therapy. ASQ-3 scores were indicative of no/low developmental risk in 16/37 (43.2%), isolated gross-motor delay consistent with spinal muscular atrophy phenotype in 8/37 (21.6%), isolated non-gross-motor delay in 3/37 (8.1%), and global developmental delay (≥2 domains) in 10/37 (27.0%). The majority of children (21/24, 87.5%) screened negative on Modified Checklist for Autism in Toddlers Revised (M-CHAT-R), indicating low risk of autism spectrum disorder. Almost one-third (32.4%) of parents reported high levels of distress. Factors associated with better developmental performance included three *SMN2* copies, diagnosis through newborn bloodspot screening and clinical silent status, absence of bulbar dysfunction, higher motor function at the time of initiation of SMN-augmenting therapy, parental well-being (absence of mental health condition and no distress) and parental attainment of tertiary education. An absence of a mental health condition in parents and three *SMN2* copy genotype in the child were identified as the strongest predictors of no/low developmental risk, with odds ratios of 4.7 and 1.4, respectively. The study findings demonstrate diverse neurodevelopmental profiles in treated children with/at-risk of spinal muscular atrophy type 1 associated with the magnitude and duration of SMN deficiency. The SMN-associated neurodevelopmental disorders may be amenable to modification by targeting bioecological factors of health. Namely, newborn screening and expedient initiation of SMN-augmenting therapies are central to targeting the neurodevelopmental window in children with/at-risk of spinal muscular atrophy type 1. Best practice includes the incorporation of proactive developmental screening for all children with/at-risk of spinal muscular atrophy type 1, with an integrated model of psychosocial support provided for families.

## Teaser

Balaji *et al*. evaluated neurodevelopment in children with/at-risk of severe spinal muscular atrophy type 1, highlighting diverse outcomes influenced by survival motor neuron 2 (*SMN2*) copies, diagnostic modality, and parental well-being, within a bioecological model. Their findings support early diagnosis and treatment, proactive developmental screening and integrated psychosocial support for affected families.

## Graphical Abstract

**Graphical Abstract:**
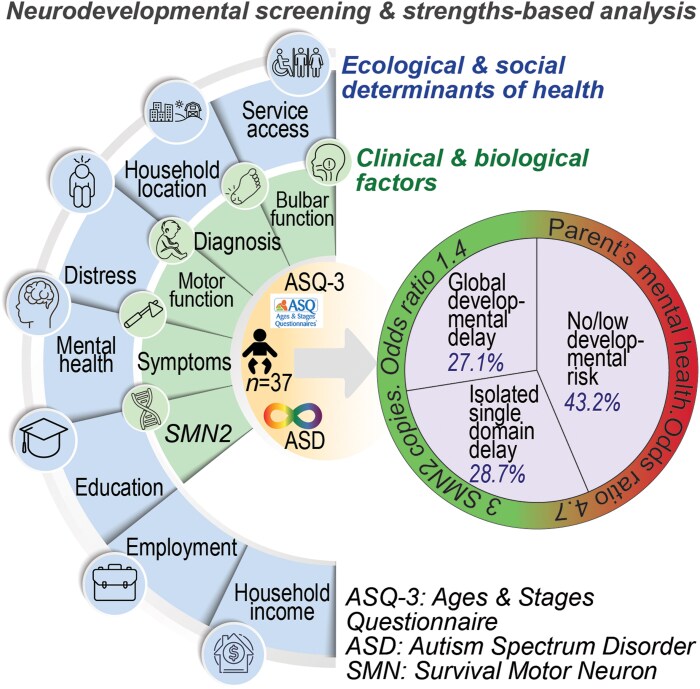

## Introduction

Genetic therapies that augment survival motor neuron (SMN) protein levels have transformed survival and motor function in children with spinal muscular atrophy (SMA). These include nusinersen and risdiplam, *SMN2* splicing modifiers and onasemnogene abeparvovec-xioi, a gene transfer therapy that uses an adeno-associated virus vector to deliver an SMN transgene. The role of SMN beyond the motor neuron is relevant in emergent phenotypes, with neuropathological data showing declining levels in the human cortex from the second trimester through post-natal periods.^1,2^ In treatment-naïve children with the severe, infantile-onset form of the condition (SMA type 1), pronounced bulbar and motor impairments influence marked deficits in the domains of communication,^3^ expressive language,^4^ spatial location,^5^ and speech.^6^ Improvements in speech acquisition and communication skills have been reported in children with SMA type 1 treated post-symptomatically,^7,8^ yet delays in cognition and communication continue to be identified.^9-11^

Newborn bloodspot screening (NBS) programmes have enabled early identification and treatment, associated with enhanced motor function outcomes.^12^ Among those diagnosed with SMA through NBS, individuals with two *SMN2* will typically develop, or have, clinical manifestations of SMA type 1, while the presence of three *SMN2* is predictive of this early-onset severe phenotype for 15% with the genotype.^13^ The developmental and communication profiles of affected children have been reported by a few studies, and to date, findings across these limited cohorts suggest that cognitive performance does not reach that of their typically developing peers.^9,11,14,15^ Thus, the role of SMN deficiency in brain development among children with or at-risk of SMA type 1 requires further investigation to inform best practice.

Whilst studies have described an emerging clinical landscape that suggests a more nuanced and complex spectrum of neurodevelopmental outcomes in children with infantile-onset SMA, the potential risk and protective factors within a bioecological model of health have not been assessed.^16^ The interaction between biological (including genetic) factors and social determinants of health may variably contribute to developmental outcomes in children with chronic conditions.^17^ Measuring the social determinants of health in other chronic conditions such as cerebral palsy has been imperative to mitigate inequities in health care access where the availability of robust medical, support and social care services tend to vary inversely with the needs of the given population. This is particularly important in the context of SMA, where SMN-augmenting therapies have transformed motor outcomes. For families, this knowledge fosters informed decision-making about therapies and education, while addressing expectations and emotional needs. For services, it facilitates the design of comprehensive care frameworks that align with the unique developmental challenges and strengths in children with SMA. However, gaps persist between emerging neurodevelopmental data and the challenges encountered in integrating these findings into clinical practice.

Formal developmental assessments are the gold standard for evaluating developmental outcomes and have been utilized in studies of children with SMA type 1 to date. However, they are time-consuming, often performance-based and may not be feasible in clinical practice due to limited resources, lengthy waiting lists and variable engagement from families. Developmental screening tests, in contrast, can provide a pragmatic and actionable alternative, enabling early identification and facilitating timely intervention for those at developmental risk. Prior studies denote the predictive value and utility of the parent-completed Ages and Stages Questionnaire (ASQ) as an effective screening tool for developmental delays in children aged 1–66 months, demonstrating strong psychometric properties, including internal consistency and test-retest reliability, between typically developing children and those with severe delays.^18^

In response to feedback from families, we designed the present study in line with their preferences, needs and values and primarily aimed to evaluate the neurodevelopmental profile of children with or predicted to have a SMA type 1 phenotype, by adopting strengths-based approach to data analysis. The secondary aim was to identify bioecological factors that could influence neurodevelopmental outcomes.

We hypothesized that in this condition, a SMN-associated neurodevelopmental disorder occurs in the context of SMN deficiency in the developing brain, which may be amenable to modification through early diagnosis and treatment. We postulated that ecological factors, including sociodemographic and parental mental health, could also determine developmental outcomes for affected children.

## Methods

### Study design and participants

This single-centre cross-sectional study followed the Strengthening the Reporting of Observational Studies in Epidemiology (STROBE) reporting guideline. The study population comprised infants and children with SMN-related SMA and their parents/caregivers. Included were children with genetically confirmed SMA (homozygous deletion of exon 7 in *SMN1*) with infantile-onset SMA (type 1, clinically manifest before age 6 months) or clinically silent SMA and a genotype of ≤3 *SMN2* at diagnosis, conferring the potential of SMA type 1, if untreated. Children outside the age range of 1 to 66 months, corresponding to the applicability of ASQ-3, were excluded.^19^ Children with SMA with additional diagnoses that could independently affect neurodevelopment, such as cerebral palsy, prematurity <32 weeks at birth, epilepsy or concurrent chromosomal disorder, were excluded. Ethics approval was obtained from the Sydney Children’s Hospitals Network Research Ethics Committee, 2023/ETH02032.

### Recruitment

Recruitment occurred from 1 January 2024 to 31 July 2024. Potential participants were identified from the Sydney Children’s Hospitals Network (SCHN) SMA clinical database, which comprised infants and children with SMA who had received diagnosis, administration of SMN-augmenting therapies and/or clinical care at SCHN (a diagnostic and treatment flagship centre for children with SMA within New South Wales, Australian Capital Territory and some parts of the Northern Territory). Study invitation and participant information sheet were provided to parents in person during hospital visits or via email. Parents were contacted thrice, with failure to respond after this point deemed as lost to contact. All parents/caregivers provided written informed consent. Parents could complete questionnaires either during routine hospital appointments, at home and return via email, or with an investigator during a phone interview, according to their preference. We organized interpreters to assist non-English-speaking participants.

### Outcome measures

A questionnaire battery, including purposively designed items (Supplementary Table 1, pages 1–2), was developed by a multidisciplinary team representing neurology, developmental paediatrics, behavioural sciences, and allied health to capture the biological and clinical characteristics of the child and the sociodemographic and clinical characteristics of the caregiver.

*Biological and clinical characteristics of the child* with SMA were collated from medical records and included age, sex, *SMN2* copy number, modality of diagnosis (NBS/clinical referral with symptoms), age and status at initiation of SMN-augmenting therapy (clinically silent or manifest), and motor function as assessed by the Children’s Hospital of Philadelphia Infant Test of Neuromuscular Disease (CHOP-INTEND) score. Current bulbar function was assessed by the Paediatric Functional Oral Intake Scale (p-FOIS) or Children’s Eating and Drinking activity Scale (CEDAS), an observer rating scale of functional oral intake and amounts, with scores ranging from 1–6, lower scores indicating more feeding and swallowing difficulties.^20^ We also asked parents to report information about their child’s enrolment in the National Disability Insurance Scheme (NDIS, a government-funded programme that provides funding for support and services of Australians with disability) via purposively designed items.

*Parent/caregiver sociodemographic and clinical characteristics* included age, sex, highest educational attainment, employment status, gross annual household income, postcode of residence, history of developmental, learning or mental health conditions. Gross annual household income was consolidated into two groups, <$34 999 and >$35 000, based on Melbourne Institute’s Poverty Lines, Australia,^21^ marking relative socioeconomic disadvantage. The residential postcode was categorized into metropolitan (MM1), regional (MM2), rural (MM3-4) and remote (MM5-7) areas according to the Modified Monash Model.^22^ A psychological distress scale using the *Kessler-10 scale (K10)* was used to evaluate parent/caregiver well-being.^23^ The K10 consists of 10 questions about emotional experiences in the previous four weeks, and participants responses range from 1 ‘none of the time’ to 5 ‘all of the time’. Total scores, calculated by summing a participant’s responses, ranged from 10 to 50, with higher scores indicating higher psychological distress, ranging from mild to severe. Participant responses were compiled into two levels of distress: no distress (score ≤19) and presence of distress (>20).^23^

*Developmental outcome measures* were assessed using the validated parent-reported *Ages and Stages Questionnaires® Third Edition (ASQ-3™*).^24^ It covers five key developmental domains: gross motor, fine motor, communication, problem-solving and personal-social, with 30 questions over the five domains. This is a tool to screen and monitor development and identify infants and young children in need of further developmental assessment. Scores ≥2 standard deviations (SD) below the mean in motor or cognitive domains are associated with moderately increased the probability of delays in these domains, while scores within 1SD of the mean indicate moderate probability of typical development without severe delay.^18^ Item scoring is categorized as ‘yes’ (scored 10), ‘sometimes’ (scored 5) or ‘not yet’ (scored 0). Scores are summated for each area; the total score in each domain was calculated and interpreted as per the ASQ user’s guide^24^ into one of the three categories:

1. ‘Requiring further assessment’, where children have scores ≤2.0 SD below age-matched population means;
2. ‘Monitor and learning activities given’, where children have scores between 1 and 2 SDs below age-matched population means; and
3. ‘On-schedule’, where children have scores ≥1SD below age-matched populations means.

For the purpose of the study, children in the first category were defined as ‘at-risk of developmental delay’ and in the latter two categories as of ‘low or no developmental risk’.^18^

To identify children at true developmental risk (beyond that of the motor manifestations of the SMA phenotype), the primary outcome was chosen *a priori* to be children with no/low developmental risk and/or delay in a single domain. With the inclusion of children with isolated single domain delay within the no/low developmental risk group, the outcome measure remained representative of the unique developmental trajectory of this population, ensuring its clinical relevance and sensitivity to the disease phenotype and therapeutic response.

Secondary outcomes included assessment of risk of autism spectrum disorder (ASD) using the *Modified Checklist for Autism in Toddlers, revised (M-CHAT-R)™* (20 items, parent-reported, 18–48 months). Each response is marked ‘yes’ or ‘no’ and the responses summated as per the scoring algorithm and categorized as low-risk (0–2), medium-risk (3–7) and high-risk (8–20) (Supplementary Fig. 1, page 3).

### Data analysis

Descriptive statistics [mean, standard deviation (SD), median and interquartile range (IQR)] were used to report the sociodemographic and clinical characteristics of the cohort. Clinical characteristics and ASQ profiles were evaluated in subgroups of children with SMA, aligning with contemporary nomenclature^25^ and included (i) clinical referral and phenotype of SMA1, (ii) diagnosis through NBS and clinically manifest at initiation of SMN-augmenting therapy, (iii) diagnosis through NBS, clinically silent and two *SMN2*, and (iv) diagnosis through NBS, clinically silent and three *SMN2*. Outcome measures with missing data were computed as per the instrument manuals. Tests of association were used to compare characteristics of study participants and eligible nonparticipants (e.g. clinical status at diagnosis, age and sex). Descriptive statistics were calculated using Graphpad Prism by Dotmatics at graphpad.com, version 10.0.02. Univariate analyses, using binomial logistic regression or Fisher’s exact test as appropriate, were conducted to examine factors influencing the primary outcome. Odds ratios (ORs) with 95% confidence intervals (CIs) were calculated to assess the strength and direction of associations between the outcome and clinical variables to facilitate interpretation of clinical applicability. Statistical significance was defined as *P* < 0.05 (two-tailed). An adaptive LASSO (Least Absolute Shrinkage and Selection Operator) variable selection procedure with leave-one-out cross-validation was performed in mice package R, version 4.4.1, using the package glmnet, to identify key predictors by penalizing less significant variables while enhancing the coefficients of more relevant ones, ensuring robust model selection and regularization.^26^ Stability of the chosen predictors was investigated by bootstrap resampling with 1000 resamples.

## Results

### Study population

Of the 41 potentially eligible children with SMA, 1 family was non-contactable, 3 did not respond to the study invitation and 1 declined participation, giving a response rate of 37/41 (90.2%). There were no significant differences between participants and eligible nonparticipants in terms of sex, clinical status at initiation of SMN-augmenting therapies and *SMN2* copy numbers (χ2 = 0.99).

**Figure 1:**
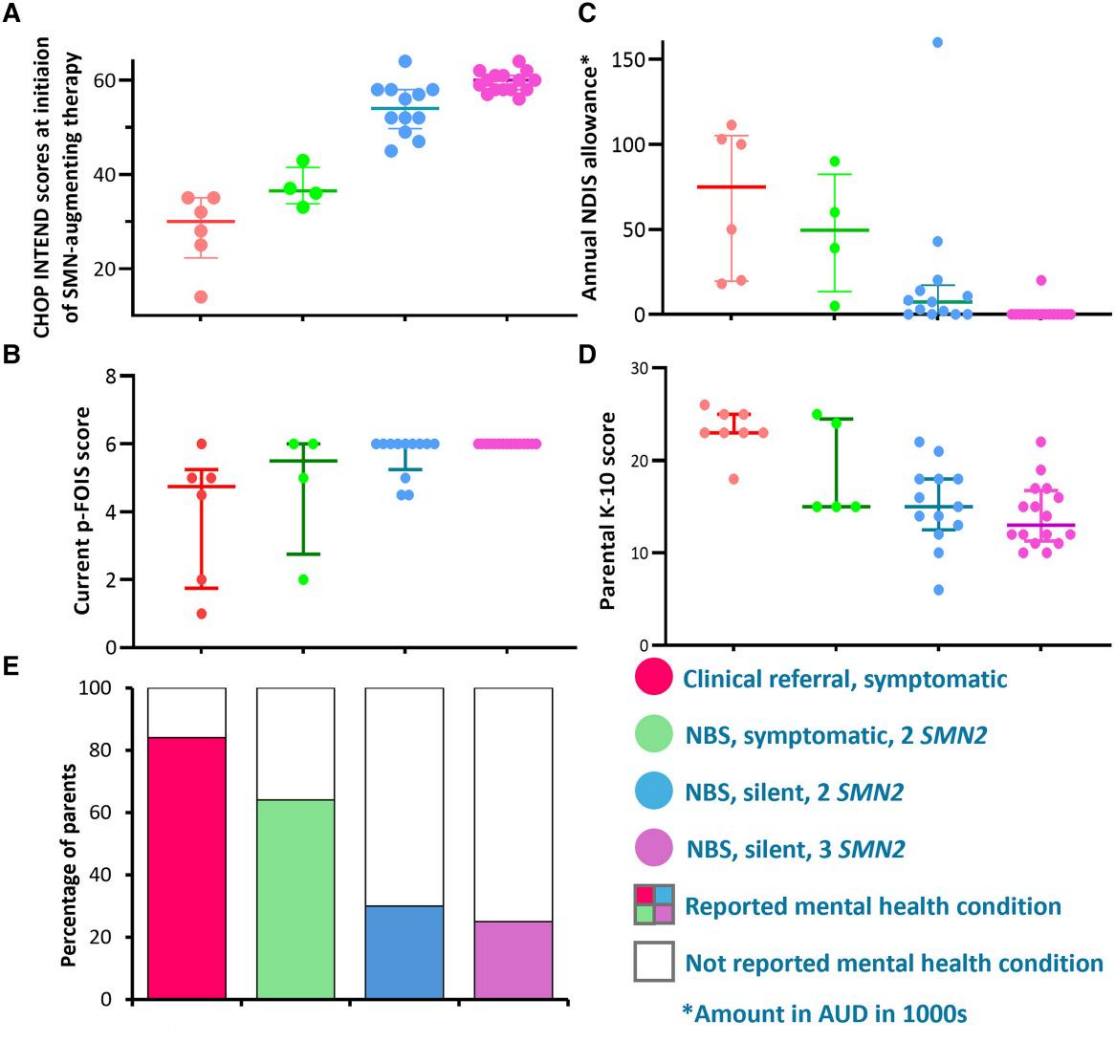
Developmental outcomes in children with SMA by diagnostic modality, clinical status and SMN2* copy number. Clinical characteristics of the cohort according to modality of diagnosis, clinical status and *SMN2* copy number at initiation of SMN-augmenting therapy (*N* = 37). (**A**) Motor function as assessed by CHOP INTEND* scores at initiation of SMN-augmenting therapy. (**B**) Current bulbar function as measured by p-FOIS/CEDAS scale*. (**C**) National Disability Insurance Scheme (NDIS) funding for supports and services. (**D**) Parental distress as measured by the Kessler-10 scale. (**E**) Reported history of parental mental health conditions. Each data point in panels A-D represents one participant. Data points in each category are presented with median (horizontal line) and interquartile ranges (IQR). *SMN2: Survival motor neuron 2 gene; CHOP INTEND: Children’s Hospital of Philadelphia Infant Test of Neuromuscular Disease; p-FOIS: Paediatric Functional Oral Intake Scale; CEDAS: Children’s Eating and Drinking Activity Scale. p-FOIS score: 1: Nothing by mouth (PG); 2: Gavage tube dependent, with minimal attempts at liquids/food (PG + PO) 3: Gavage tube dependent, with consistent intake of liquids/foods (PG + PO) 4: Total oral diet, but requiring special preparation of liquids ± compensations (PO + compensations) 4.5: Total oral diet, but requiring special preparation of solids ± compensations (PO + compensations) 5: Total oral diet, without special preparation, but with compensations (PO + compensations) 6: Total oral diet, with no restrictions relative to peers (PO).

The parent completing the questionnaires was the primary caregiver in 34/37 (91.9%) of participants (mother: 31/37, 83.8% and father: 6/37, 16.2%). The median age (IQR) of children with SMA was 43 months (26.5–53.5) (Table 1). Most children were diagnosed through newborn or prenatal screening (31/37, 83.8%), of which 4 with two *SMN2* were clinically manifest and 27 (12 with two *SMN2* and 15 with three *SMN2)* were clinically silent at initiation of SMN-augmenting therapy. Diagnosis following clinical referral after symptoms occurred for 6/37 (16.2%), all with two *SMN2*. All children in this cohort (37/37, 100%) received SMN-augmenting therapy(ies). Clinical characteristics were delineated according to modality of diagnosis, clinical status at initiation of SMN-augmenting therapy and *SMN2* copy number (Fig. 1).

**Table 1:** Sociodemographic and clinical characteristics of children with SMA and their parents/caregivers

Characteristic	Number (%)
Age of child (months)	
Median (IQR)	43 (26.5–53.5)
Age at initiation of SMN-augmenting therapy (months)	
Median (IQR)	0.8 (0.6–1.4)
Sex	
Male	17 (46)
Female	20 (54)
Respondent’s relation	
Mother	31 (83.7)
Father	6 (16.3)
Diagnosis of SMA	
Newborn or prenatal screening	31 (83.8)
Diagnosis following clinical referral with symptoms	6 (16.2)
Number of copies of *SMN2*	
2 *SMN2*	22 (59.4)
3 *SMN2*	15 (40.6)
Clinical status at initiation of SMN-enhancing therapy	
Clinically manifest	10 (27)
Clinically silent	27 (73)
Parental educational attainment	
Secondary school or less	9 (24.4)
Tertiary education and beyond	28 (75.6)
Parental history of mental health condition(s)	
None reported	20 (54.1)
Reported^a^	17 (45.9)
Parental employment status	
Employed	9 (51.4)
Unemployed	18 (48.6)
Gross annual household income from earnings or pensions^b^	
Below $34 999	10 (27.0)
$35 000 and above	24 (64.9)
Area of residence^c^	
Metropolitan	25 (67.5)
Regional/rural/remote	12 (32.4)
National Disability Insurance Scheme (NDIS)	
Approved	19 (51.3)
Ineligible/rejected/not applied	18 (48.6)

^a^Reported mental health conditions: Anxiety and depression 10 (27%), psychoses 2 (5.4%), multiple diagnoses 3 (8.1%), post-traumatic stress disorder (PTSD), post-natal depression 5 (13.5%).

^b^Preferred not to answer. ^c^Modified Monash Model:^22^ Metropolitan (MM1), regional (MM2), rural (MM3-4) and remote (MM5-7).

### Developmental characteristics on ASQ-3 of children with SMA

**Figure 2:**
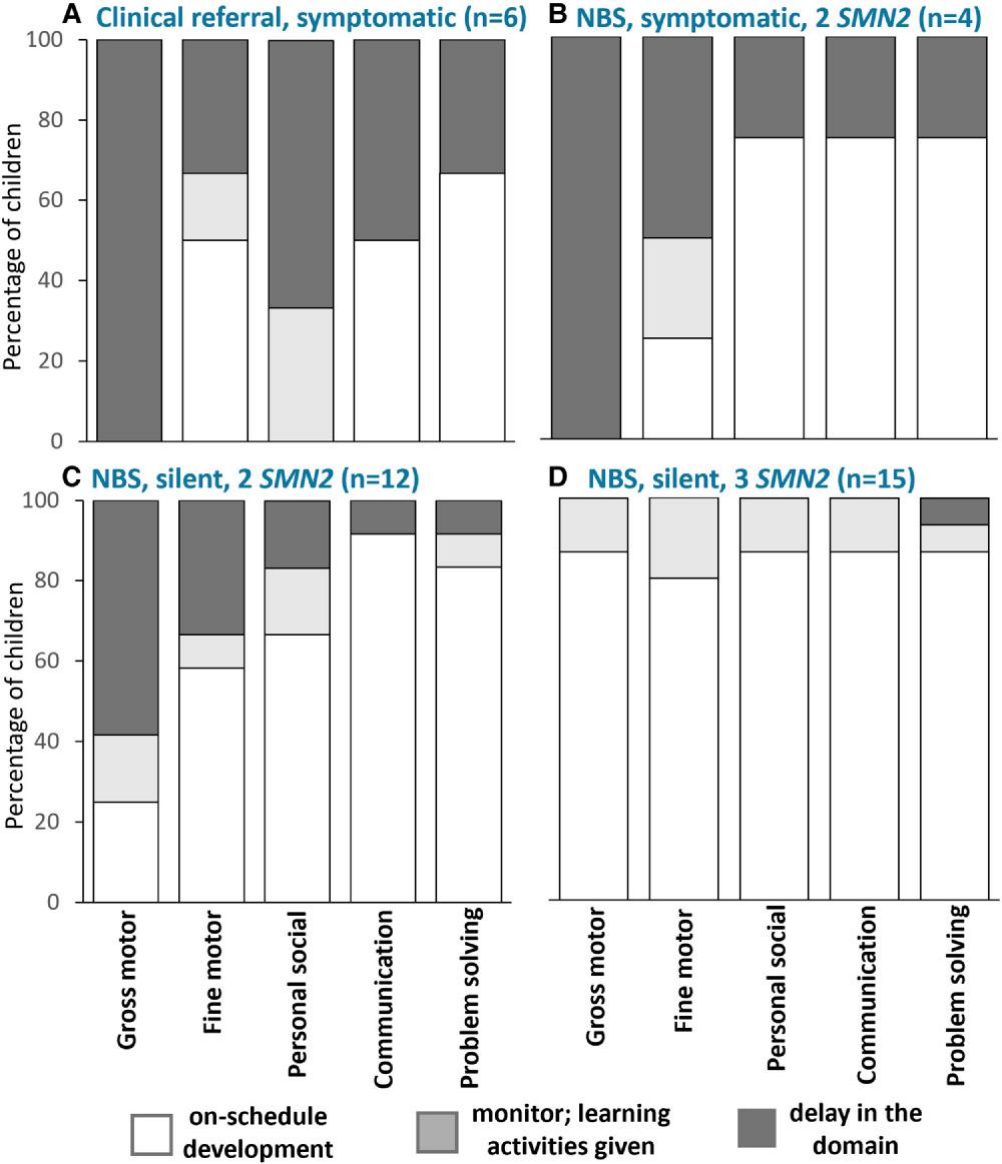
Parent-reported ASQ-3* outcomes by clinical characteristics. Parent-reported development of children with SMA as assessed by the Ages and Stages Questionnaire-3, according to clinical status at diagnosis (*N* = 37). (**A**) Development in symptomatic children, referred after symptom onset (*n* = 6). (**B**) Development in symptomatic children, diagnosed through NBS, and with two *SMN2* copies (*n* = 4). (**C**) Development in clinically silent children, diagnosed through NBS, and with two *SMN2* copies (*n* = 12). (**D**) Development in clinically silent children, diagnosed through NBS, and with three *SMN2* copies (*n* = 15). *ASQ-3: Ages and Stages Questionnaire 3rd edition. Each bar represents the percentage of children who were on-schedule (unshaded), required monitoring (light grey), or showed delay in each developmental domain (dark grey). Children who were on-schedule or required monitoring were considered to have no/low developmental risk, while those with a delay were classified as having a delay in the domain.

Of the cohort, 16/37 (43.2%) children with SMA had ‘no/low developmental risk’ (16/37, 43.2%). All of these children (16/37, 43.6%) were diagnosed through NBS and were clinically silent at initiation of SMN-augmenting therapy; this group included those with development on-schedule (8/37, 21.6%), comprising 6/15 (40%) with three *SMN2* and 2/22 (9.1%) with two *SMN2* and the other ‘close to cutoff’ of 8 children (8/37, 21.6%), the majority (7/16, 37.5%) having three *SMN2*. Among those with clinically manifest SMA, isolated gross-motor delay was identified in 4/10 (40%) and delay in ≥2 domains in 6/10 (60%) (Fig. 2). Isolated delay in a single domain was found in 11/37 (27.9%) children; thus, among the cohort, 27/37 children (72.9%) demonstrated no/low developmental risk or isolated delay in a single domain, fulfilling the primary outcome measure.

Developmental strengths within the cohort were in the domains of communication where (32/37, 86.4%) of children were at no/low developmental risk, followed by problem-solving (31/37, 83.8%), personal-social (30/37, 81.1%) and fine motor (29/37, 78.4%) skills (Supplementary Table 2, pages 4–5). Delay in ≥2 domains was identified in 10/37 (27%) children, and this subgroup included 6/37 (16.2%) children who were clinically symptomatic at initiation of SMN-augmenting therapy, all requiring tube feeding ± compensations (Supplementary Table 3, pages 6–7). ASQ scores indicative of delay in all developmental domains were reported in 3/37 (8.1%). No child diagnosed through NBS had ASQ scores indicative of isolated delay in communication, while for the 3 children with isolated delay in personal-social or problem-solving, parents reported a lack of opportunity to try the skill within their cultural context.

For children at no/low developmental risk or isolated delay in a single domain, there were significant associations with biological and clinical factors including three *SMN2* copies (OR, CIs not reportable, *P* = 0.002), NBS for diagnosis (OR: 8.33, CI: 1.42–47.95, *P* = 0.035) and clinically silent status at initiation of SMN-augmenting therapy (OR: 10.27, CI: 1.94–54.27 *P* = 0.006). Ecological variables associated with no/low developmental risk or isolated delay in a single domain included parental attainment of tertiary education (OR: 12.00, CI: 2.05–53.34, *P* = 0.0053), no reported mental health condition in parent/carer (OR: 21.38, CI: 2.85–243.9, *P* = 0.0020) and no parental distress (OR: 18.67, CI: 3.10–86.40, *P* = 0.0011) on univariate analyses (Table 2). No significant association was observed with parental employment, gross annual income or residential postcode.

**Table 2:** Univariate analyses of factors associated with the primary outcome: ASQ^a^ scores indicative of no/low developmental risk or isolated delay in one domain

Factor	Unadjusted OR	95% CIs	*P*-value
Clinical and biological factors in children with SMA			
Sex			
(girl versus boy)	1.25	0.29, 5.35	0.764
*SMN2* copies			
(3 *SMN2* versus 2 *SMN2*)	NR^b^	NR^b^	**0.002**
Modality of diagnosis			
(NBS^a^ versus diagnosis following referral with symptoms)	8.33	1.42, 47.95	**0.035**
Clinical status at initiation of SMN-augmenting therapy			
(silent versus manifest)	10.27	1.94, 54.27	**0.006**
Motor function, as assessed by CHOP INTEND^a^ scores at initiation of SMN-augmenting therapy	1.09	1.02, 1.17	**0.010**
Current feeding and swallowing function, as assessed by p-FOIS/CEDAS^a^ score	7.00	1.51, 32.44	**0.013**
Sociodemographic factors of parent respondents			
Educational attainment			
(tertiary and beyond versus secondary and below)	12.00	2.05, 53.34	**0.0053**
Distress			
{low score on K-10 (<20) versus high score on K-10 (≥20)}	18.67	3.10, 86.40	**0.0011**
Reported history of mental health condition(s)			
(none reported versus reported^c^)	21.38	2.85, 243.9	**0.0020**
Current employment status			
(employed versus not employed)	3.39	0.81, 13.69	0.15
Gross household annual income^d^			
(>$35 000 versus <$34 999)	4.40	0.84, 17.71	0.094
Postcode of residence^e^			
(metropolitan versus regional/rural/remote)	2.86	0.60, 14.48	0.23

The reference group was identified a priori based on a strengths-based approach to data analysis. The bold values represent statistically significant results (*P* <0.05), indicating a clinically meaningful association based on unadjusted odds ratios and their 95% confidence intervals from logistic regression analysis.

^a^ASQ, Ages and Stages Questionnaire; NBS, Newborn bloodspot screening; CHOP INTEND, Children's Hospital of Philadelphia Infant Test of Neuromuscular Disease; p-FOIS, Paediatric Functional Oral Intake Scale; CEDAS, Children's Eating and Drinking Activity Scale.

^b^NR, not reported due to a participant count of 0 in one of the comparison groups, which resulted in infinite OR (Odds ratio) and non-estimable CIs (Confidence Intervals).

^c^Reported mental health conditions: Anxiety and depression 10 (27%), psychoses 2 (5.4%), multiple diagnoses 3 (8.1%), post-traumatic stress disorder (PTSD), post-natal depression 5 (13.5%).

^d^Reported by 34 families only.

^e^Modified Monash Model:^22^ Metropolitan (MM1), regional (MM2), rural (MM3-4) and remote (MM5-7).

**Figure 3:**
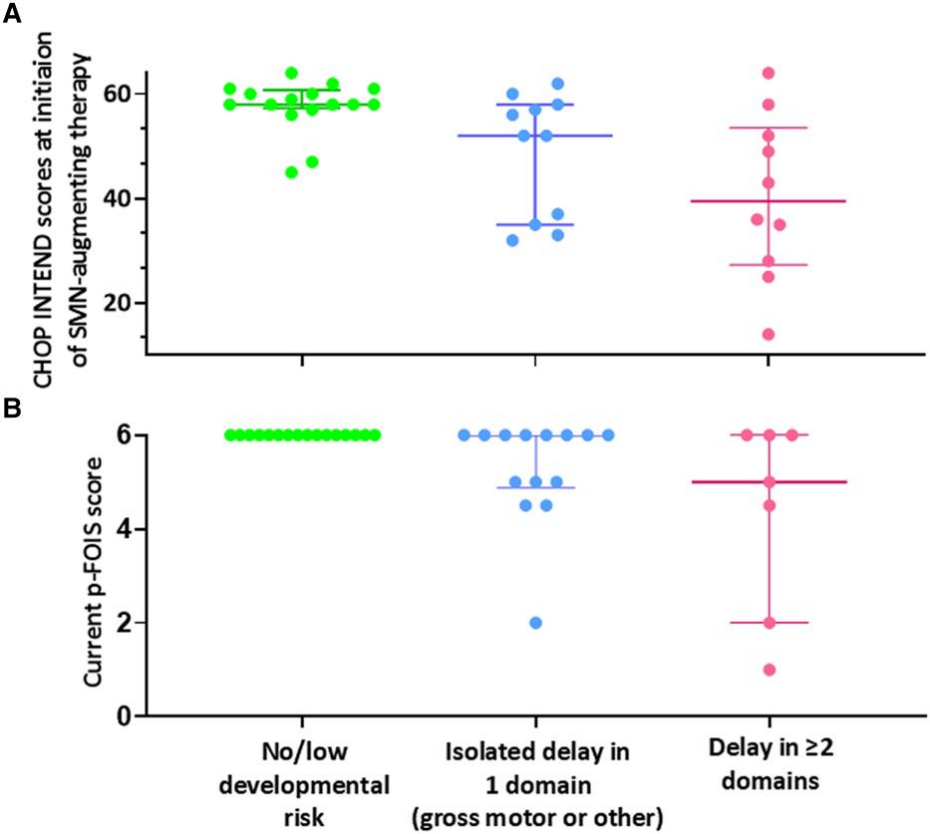
**Motor and bulbar function across developmental categories in SMA.** Distribution of motor function scores at initiation of SMN-augmenting therapy and current bulbar function scores across developmental categories in children with SMA (*n* = 37). (**A**) CHOP-INTEND* B: p-FOIS/CEDAS*. Each data point represents one participant. Data points in each category are presented with median (horizontal line) and interquartile ranges (IQR). *CHOP INTEND: Children’s Hospital of Philadelphia Infant Test of Neuromuscular Disease; p-FOIS: Paediatric Functional Oral Intake Scale; CEDAS: Children’s Eating and Drinking Activity Scale. Logistic regression analysis showed that no/low developmental risk or isolated delay in a single domain was significantly associated with (**A**) higher CHOP-INTEND scores (OR: 1.09, 95%CIs: 1.02, 1.17, *P* = 0.010) and (**B**) better current bulbar function (OR: 7.00, 95%CIs: 1.51, 32.44, *P* = 0.013). OR, Odds ratio; CIs, Confidence Intervals.

Children with no/low developmental risk or isolated delay in a single domain had significantly higher motor function at initiation of SMN-augmenting therapy (OR: 1.09, CI: 1.02, 1.17, *P* = 0.010) and current bulbar function (OR: 7.00, CI: 1.51, 32.44, *P* = 0.013) than those at-risk of developmental delay. For one-point increase each in CHOP-INTEND score at initiation of therapy and current p-FOIS score, the odds of being in no/low developmental risk category increased by 1.1 times and 7.0, respectively (Fig. 3).

Clinical and biological factors in children and sociodemographic factors of parents were included in the LASSO procedure to select those most predictive of the primary outcome of no/low developmental risk or isolated delay in a single domain relative to those with a risk of delay. Absence of parental mental health concerns (LASSO OR: 4.73) and three *SMN2* (LASSO OR: 1.44) together predicted a higher probability of a child having no/low developmental risk or isolated (single domain) developmental delay. Bootstrapping to assess the stability of the variable selection procedure found that parental mental health was selected in 100% of cases and *SMN2* in 73.3% of cases, indicating their strong relationship with no/low developmental risk; K10 (binary) was selected in only 1.5% of the resamples, suggesting its limited relevance as a predictor.

### Risk of autism Spectrum disorder

Among the 24 children assessed using M-CHAT-R™, scores indicative of a low risk of ASD were ascertained in 21/24 (87.5%) children. The 3/24 (12.5%) children with M-CHAT-R scores indicative of high-risk of ASD were all boys, clinically manifest at diagnosis and had two *SMN2* copies. Diagnostic evaluation confirmed level 2 or level 3 ASD in these children as per Diagnostic and Statistical Manual 5 criteria (DSM-5-TR).

## Discussion

This study has identified heterogeneous developmental profiles among children with SMA who inherit severe genotypes, ranging from those with a typical developmental trajectory to others with isolated motor delay in keeping with the motor effects of SMN deficiency. The study highlights that nearly one-fourth of children with or predicted to have infantile-onset SMA are at risk of delays in two or more developmental domains.

At the clinical interface, parents want to understand whether developmental differences for their child are part of the biology of SMA or influenced by environmental factors. This study has generated new knowledge, determining that developmental outcomes in SMA may be strongly and concomitantly driven by biological factors alongside key psychosocial determinants of health, suggesting that the developmental sequelae of this monogenic condition may have the potential to be modified by targeted early interventions.

This study builds on the current evidence base for an integral role of SMN in CNS development, demonstrating its manifestations as SMN-associated neurodevelopmental disorders. Here, the duration (associated with disease onset and clinical status at initiation of SMN-augmenting therapy) and magnitude of SMN deficiency (associated with *SMN2* copy number) appear to be significantly associated with developmental outcomes, with *SMN2* copy number the strongest biological modifier of development in children with SMA. This clinical data augments findings from preclinical and histopathological studies, which have underlined the specific function of SMN in axogenesis, axonal sprouting, neuronal migration and differentiation in the central nervous system.^27,28^ Further, post-mortem histopathological data from untreated children with SMA have shown the effects of SMN depletion on brain structures, including the thalami, basal ganglia, temporal and frontal cortices, hippocampi and cerebellum, with progression in changes over time.^1,29^

The evidence from this study underlines the concept that SMA is a neurogenetic emergency for children predicted to have infantile-onset SMA, not only from the perspective of preserving the motor unit pool, but also the developing brain. Expedient SMN restoration within a neurodevelopmental window, facilitated by NBS and timely access to therapies, may optimize future developmental outcomes.

Whilst prior studies have shown challenges with communication in children with SMA,^8^ and improvements in speech acquisition post-treatment,^7^ the extent of (appendicular) muscle and bulbar denervation and the impact on development in this domain have not been interrogated. Our study fills this knowledge gap and notes that severe muscle weakness and bulbar dysfunction affect developmental gains, with the odds of having no/low developmental risk one and seven times higher for every 1-point increase in assessment scores within these two domains.^10,15,30^ A proactive model of care with access to early (allied therapy) intervention and integrated support to target bulbar and muscle health and function (aligning with targeted and goal-specific interventions in other neurodevelopmental disorders such as cerebral palsy) may support wider neurodevelopmental processes such as communication, problem-solving and personal-social skills which are highly valued by families to promote functional independence, participation and inclusion.^31^ Longitudinal monitoring and further research to determine the effectiveness of interventions is needed.

Concomitant with biological drivers of development, our findings determine that psychosocial determinants of health, as applied to other chronic conditions, are also relevant to children with SMA.^32^ Notably, an absence of parental mental health conditions was strongly associated with no/low developmental risk in the child, providing evidence and opportunity for early, comprehensive and family centric psychological support to be embedded within a model of care that may influence developmental outcomes over the course of the lifetime for the child. Furthermore, the prevalence of parental distress in our study was three times higher than that observed in the general adult population,^33^ highlighting the potential impact of caregiving demands on mental health, aligning with similar outcomes in other neurodevelopmental conditions. This has translational implications necessitating targeted surveillance and support.

Other key social determinants of health, including region/residential postcode within Australia, parental income and employment (which have been identified as significant factors in other neurodevelopmental conditions), were not associated with or predictive of developmental outcomes in our study. This is postulated to be secondary to the impact of NBS for SMA and provision of medicare facilities that provide equitable access to diagnosis and early treatment, mitigating these sociodemographic factors and thus, their influence on developmental outcomes. In the context of shifting diagnostic and treatment approaches for SMA, a spectrum of SMN-associated neurodevelopmental disorders in children with/at-risk of SMA type 1 is evident, with biological and ecological drivers playing key roles in outcomes. This study also highlights the critical window for neurodevelopmental protection, reinforcing the concept that ‘*time is brain***’** during key developmental stages.

### Strengths, limitations and future directions

Our study used a novel strengths-based data analysis as a unique approach to development within SMA, aligning with the preferences of families to identify factors associated with developmental preservation and optimization. This changes the paradigm within the field from a deficit-based narrative to a strengths-based one, fostering family engagement and collaboration in the child’s developmental journey. The findings generate knowledge on the potential to identify factors amenable to modification that may support neurodevelopmental outcomes, including the requirement for a psychosocial model of care that supports parental well-being.

The ASQ-3 is an instrument used for screening or monitoring development, however, can be influenced by opportunities to try the skill or cultural factors and as such is limited to those children who are exposed to and practice these pre-specified tasks.^34^ While parental responses have limitations including recall and reporting biases (in either direction with under and over reporting of developmental abilities), ASQ-3 has been widely recognised as a valid guide to aid decision-making regarding the need to refer to speciality services for a performance-based assessment or surveillance.^35^ The use of a parent-reported screening tool instead of a performance-based assessment feeds directly into the presentation of children with SMA, where variabilities arise in the latter dependent on levels of fatigue, interest and understanding of instructions. Additionally, a parent-reported tool provides a broader view of development across environments as pertains to everyday function. Also, the ASQ-3 is widely used in clinical practice and is thus easily translated into the scope of developmental surveillance within current health services, with clear criteria on when to continue developmental surveillance and red flag triggers for initiation of early intervention and formal developmental assessment at a suitable time. Our clinically grounded approach to data analysis may mitigate this potential limitation of imprecision in the primary outcome.

Whilst the cohort was established from a single centre, it successfully captured the changing diagnostic and therapeutic approaches and a diverse range of sociodemographic characteristics. Study strengths also include the robust response rate and sample size for a rare disease; however, several limitations warrant discussion. The influence of cultural identification as a key social determinant of health was not assessed in this study. Recruitment across a single health system may limit generalisability, particularly for those centres without established NBS programmes and/or low-income settings. The identification of three *SMN2* copies and parental mental health concerns through the LASSO procedure provides potential avenues for future research; however, the large estimates derived from a small sample size and the binary outcome distribution warrant caution in interpretation. Future collaborative multicentre studies with increased power for statistical analyses would enable a comprehensive understanding of the diverse neurodevelopmental profiles of children with SMA, their trajectories and the causal and potentially modifiable factors. Our study relied on parental self-reports of mental health conditions/diagnoses, which is another source of potential bias, possibly leading to underreporting. Due to the impact of parental mental health on neurodevelopment observed in this study, future research could benefit from the use of other methodologies, including validated condition-specific measures and mental health diagnostic interviews, as well as a more nuanced examination of parent and family mental health history. Finally, the cross-sectional design restricted the ability to assess causality and examine longitudinal changes that would result from interventions.

Future research should focus on developing a comprehensive model of SMA that includes not only motor development but also executive functions, behaviours, and well-being as a continuum from infancy through adolescence. Whilst this study provides a snapshot of development in children with SMA type 1, the incorporation of developmental outcomes from real-world populations through data registries remains imperative to understand the longitudinal effects of treatment and active pursual of standards of care.^36^ The developmental screening tool used in this study gives an overview of development, however a more comprehensive assessment of executive function for school aged children with SMA type 1 is important to establish areas of strength and challenges that require educational support and planning. Whilst this study has focussed on development in children with SMA type 1, children with later onset forms of SMA are known to have challenges with behaviour, regulation and well-being, which require characterisation and intervention in future studies. The development of SMA-specific developmental scales may help to capture the unique challenges faced by children with SMA and enable tailored assessments and interventions.

### Recommendations for clinical practice

The study establishes the evidence base for a streamlined and efficient NBS model of care to shorten the time to therapeutic intervention. In a health ecosystem with finite resources, incorporating developmental screening into clinical practice may help to better identify children at risk of developmental delays and provide early interventions. Namely, targeted strategies to maintain and optimize bulbar and muscle function may have downstream consequences on overall development. Whilst biological drivers are important, parental mental health screening and a triage system for appropriate support and referral align with best practice and are warranted to mitigate the significant effects of this social determinant of health on developmental outcomes.

## Supplementary Material

fcaf272_Supplementary_Data

## Data Availability

Data collected for this study, including individual de-identified participant data and a data dictionary defining each field in the set, may be available to suitably qualified researchers through reasonable requests. Applicants willing to receive the data should apply within 12 months after the manuscript has been published in print and should demonstrate that the proposed use of the data has been approved by an independent review committee identified for this purpose. The data request should then be sent to the corresponding author, and de-identified data will be shared with a signed data access agreement. The full R code used for regression analyses, including adaptive LASSO and bootstrap procedures, is provided in Supplementary File 1, pages 8–11.

## References

[1] Ramos DM, d’Ydewalle C, Gabbeta V, et al Age-dependent SMN expression in disease-relevant tissue and implications for SMA treatment. J Clin Invest. 2019;129(11):4817–4831. 31589162 10.1172/JCI124120 PMC6819103

[2] Towfighi J, Young RS, Ward RM. Is Werdnig-Hoffmann disease a pure lower motor neuron disorder? Acta Neuropathol. 1985;65:270–280. 3976363 10.1007/BF00687008

[3] Hoshi Y, Sasaki C, Yoshida K, et al Milestones for communication development in Japanese children with spinal muscular atrophy type I. J Health Sci. 2017;14:115–120.

[4] Ball LJ, Chavez S, Perez G, et al Communication skills among children with spinal muscular atrophy type 1: A parent survey. Assist Technol. 2021;33(1):38–48. 30945993 10.1080/10400435.2019.1586788

[5] Polido GJ, Barbosa AF, Morimoto CH, et al Matching pairs difficulty in children with spinal muscular atrophy type I. Neuromuscul Disord. 2017;27(5):419–427. 28302390 10.1016/j.nmd.2017.01.017

[6] Zappa G, LoMauro A, Baranello G, et al Intellectual abilities, language comprehension, speech, and motor function in children with spinal muscular atrophy type 1. J Neurodev Disord. 2021;13(1):9. 33530934 10.1186/s11689-021-09355-4 PMC7856807

[7] Al-Zaidy S, Pickard AS, Kotha K, et al Health outcomes in spinal muscular atrophy type 1 following AVXS-101 gene replacement therapy. Pediatr Pulmonol. 2019;54(2):179–185. 30548438 10.1002/ppul.24203 PMC6590370

[8] Pane M, Palermo C, Messina S, et al An observational study of functional abilities in infants, children, and adults with type 1 SMA. Neurology. 2018;91(8):e696–e703. 30045959 10.1212/WNL.0000000000006050 PMC6107268

[9] Kölbel H, Kopka M, Modler L, et al Impaired neurodevelopment in children with 5q-SMA-2 years after newborn screening. J Neuromuscul Dis. 2024;11:143–151. 37927272 10.3233/JND-230136 PMC10789341

[10] Steffens P, Weiss D, Perez A, et al Cognitive function in SMA patients with 2 or 3 SMN2 copies treated with SMN-modifying or gene addition therapy during the first year of life. Eur J Paediatr Neurol. 2024;51:17–23. 38772209 10.1016/j.ejpn.2024.05.002

[11] Ngawa M, Dal Farra F, Marinescu AD, Servais L. Longitudinal developmental profile of newborns and toddlers treated for spinal muscular atrophy. Ther Adv Neurol Disord. 2023;16:17562864231154335. 36846472 10.1177/17562864231154335 PMC9944336

[12] Kariyawasam DST, D'Silva AM, Vetsch J, Wakefield CE, Wiley V, Farrar MA. “We needed this”: Perspectives of parents and healthcare professionals involved in a pilot newborn screening program for spinal muscular atrophy. EClinicalMedicine. 2021;33:100742. 33842861 10.1016/j.eclinm.2021.100742 PMC8020144

[13] Calucho M, Bernal S, Alías L, et al Correlation between SMA type and SMN2 copy number revisited: An analysis of 625 unrelated Spanish patients and a compilation of 2834 reported cases. Neuromuscul Disord. 2018;28(3):208–215. 29433793 10.1016/j.nmd.2018.01.003

[14] Buchignani B, Cicala G, Cumbo F, et al Communicative development inventory in type 1 and presymptomatic infants with spinal muscular atrophy: A cohort study. Arch Dis Child. 2024;109(5):395–401. 38290776 10.1136/archdischild-2023-326613

[15] Masson R, Brusa C, Scoto M, Baranello G. Brain, cognition, and language development in spinal muscular atrophy type 1: A scoping review. Dev Med Child Neurol. 2021;63(5):527–536. 33452688 10.1111/dmcn.14798

[16] Bronfenbrenner U . Ecological systems theory (1992). Making human beings human: Bioecological perspectives on human development. Sage Publications Ltd; 2005:106–173.

[17] Woolfenden S, Galea C, Smithers-Sheedy H, et al Impact of social disadvantage on cerebral palsy severity. Dev Med Child Neurol. 2019;61(5):586–592. 30221759 10.1111/dmcn.14026

[18] Muthusamy S, Wagh D, Tan J, Bulsara M, Rao S. Utility of the ages and stages questionnaire to identify developmental delay in children aged 12 to 60 months: A systematic review and meta-analysis. JAMA Pediatr. 2022;176(10):980–989. 36036913 10.1001/jamapediatrics.2022.3079 PMC9425289

[19] Squires J, Twombly E, Bricker DD, Potter LW. ASQ-3 User's guide. Paul H. Brookes Pub.; 2009.

[20] Weststrate H, Stimpson G, Thomas L, et al Evolution of bulbar function in spinal muscular atrophy type 1 treated with nusinersen. Dev Med Child Neurol. 2022;64(7):907–914. 35103306 10.1111/dmcn.15171 PMC9306995

[21] Research MIoAEaS . Poverty Lines Australia. University of Melbourne. Accessed September 2024. https://melbourneinstitute.unimelb.edu.au/publications/poverty-lines

[22] Model MM . Australian Government, Department of Health and Aged Care. 2024. Accessed 2024. https://www.health.gov.au/topics/rural-health-workforce/classifications/mmm#:∼:text=The Modified Monash%20Model %28MMM%29 is how we,major%20city and MM 7 is very remote

[23] Use of the Kessler Psychological Distress Scale in ABS Health Surveys, Australia, 2007–08. Australian Bureau of Statistics. Accessed January 2024. https://www.abs.gov.au/ausstats/abs@.nsf/Lookup/4817.0.55.001Chapter92007-08

[24] Squires J, Bricker DD, Twombly E. Ages & stages questionnaires. Paul H. Brookes Baltimore; 2009.

[25] Balaji L, Farrar MA, D’Silva AM, Kariyawasam DS. Decision-making and challenges within the evolving treatment algorithm in spinal muscular atrophy: A clinical perspective. Expert Rev Neurother. 2023;23:571–586. 37227306 10.1080/14737175.2023.2218549

[26] R Core Team . R: A language and environment for statistical computing. R Core Team. 2013.

[27] Franco-Espin J, Gatius A, Armengol J”, et al SMN is physiologically downregulated at wild-type motor nerve terminals but aggregates together with neurofilaments in SMA mouse models. Biomolecules. 2022;12(10):1524. 36291733 10.3390/biom12101524 PMC9599093

[28] Giavazzi A, Setola V, Simonati A, Battaglia G. Neuronal-specific roles of the survival motor neuron protein: Evidence from survival motor neuron expression patterns in the developing human central nervous system. J Neuropathol Exp Neurol. 2006;65(3):267–277. 16651888 10.1097/01.jnen.0000205144.54457.a3

[29] Wishart TM, Huang JP-W, Murray LM, et al SMN deficiency disrupts brain development in a mouse model of severe spinal muscular atrophy. Hum Mol Genet. 2010;19(21):4216–4228. 20705736 10.1093/hmg/ddq340 PMC2951867

[30] Harding BN, Kariya S, Monani UR, et al Spectrum of neuropathophysiology in spinal muscular atrophy type I. J Neuropathol Exp Neurol. 2015;74(1):15–24. 25470343 10.1097/NEN.0000000000000144 PMC4350580

[31] Keen R . The development of problem solving in young children: A critical cognitive skill. Annu Rev Psychol. 2011;62:1–21. 20822435 10.1146/annurev.psych.031809.130730

[32] Ostojic K, Karem I, Paget SP, et al Social determinants of health for children with cerebral palsy and their families. Dev Med Child Neurol. 2024;66(1):32–40. 37179527 10.1111/dmcn.15640

[33] K-10. Kessler Psychological Distress Scale (K10). Agency for Clinical Innovation. Accessed January 2024. https://aci.health.nsw.gov.au

[34] Guidelines for Cultural and Linguistic Adaptation of ASQ®-3 and ASQ®:SE-2. Brookes Publishing. https://agesandstages.com/wp-content/uploads/2018/03/Guidelines-for-Cultural-and-Linguistic-Adaptation-of-ASQ.pdf

[35] Mackin R, Ben Fadel N, Feberova J, et al ASQ3 and/or the Bayley-III to support clinicians’ decision making. PLoS One. 2017;12(2):e0170171. 28151969 10.1371/journal.pone.0170171 PMC5289417

[36] Balaji L, Forbes R, Cairns A, et al A contemporary analysis of the Australian clinical and genetic landscape of spinal muscular atrophy: A registry based study. Lancet Reg Health West Pac. 2024;53:101237. 39559164 10.1016/j.lanwpc.2024.101237 PMC11570865

