# Supplementary material for: Neurodevelopmental screening in children with early-onset spinal muscular atrophy in the treatment era: a strengths-based cohort study
Source: Brain Commun. 2025 Jul 21;7(4):fcaf272. doi: 10.1093/braincomms/fcaf272 (PMC12341873; doi:10.1093/braincomms/fcaf272)
Supplement: fcaf272_Supplementary_Data [file fcaf272_supplementary_data.docx]

**Supplementary Table 1. Study measures to explore participants’ sociodemographic data, parental education and mental health, and access to services in the community**

| ***Domain*** | ***Measures*** | ***Response options*** |
| --- | --- | --- |
| Sociodemographic data of child and parent | Age of child  Sex of child  Age of respondent  Sex of respondent  Postcode of residence  Gross annual household income | Below $34,999  $35,000 - $59,999  $60,000 - $99,999  $100,000 - $199,999  $200,000 - $299,999  Above $300,000  Prefer not to answer |
| Parental characteristics | Highest level of education | < year 10  Year 12  Apprenticeship/ Certificate course  University – Bachelors  Post graduate degree  Doctoral degree |
|  | Employment status | Yes/No; if yes, full-time or part-time or casual |
|  | History of developmental or learning problems in either parent? | Yes/No; if yes, which one?  Autism Spectrum Disorder  Attention Deficit Hyperactivity Disorder (ADHD)  Learning disability  Dyslexia, dysgraphia, dyspraxia  Cerebral palsy  Other (include Tourette syndrome, Fragile X syndrome, other similar conditions, if present) |
|  | Previous/current diagnosis of mental health condition in either parent? | Yes/No; if yes, which one?  Anxiety  Depression  Schizophrenia  Schizoaffective Disorder  Bipolar Affective Disorder  Borderline Personality Disorder  Other (include disorders not mentioned above) |
| Child characteristics | Attendance at school/ daycare | Yes/No  If yes, number of days/ week |
|  | How does the child mobilize indoors? | Walks independently  Walks with a gait aid  Crawls or furniture cruises around the house  Independent using a manual wheelchair  Independent using a power wheelchair  Independent using a walking aid  Not independent, needs assistance to move |
|  | How does the child mobilize in the community? | Walks independently  Walks with a gait aid  Pushes in a manual chair/ stroller  Independent using a manual wheelchair  Independent using a power wheelchair |
| Policy questions | Is your child enrolled in National Disability Insurance Scheme (NDIS)? | Yes  No  Applied, and waiting for response  Never applied  Was rejected |
|  | Does the NDIS provide you with care coordination or have you applied for care coordination? | Yes/No |
|  | If you have a current package, what is the value of your core support part of your current NDIS package? | Yes/No |
|  | Is there a therapy that you would like for your child but do not have access to? |  |
| Recommendations/ suggestions | Do you have any suggestions regarding incorporation of developmental assessments in SMA? ­­­­­­­­­­­­­­­­­­­­ |  |
|  | Would you recommend participation in questionnaires for developmental and behavioural screening to friends/other families? | Yes/No  Please explain your reasons |

**Supplementary Figure 1. Overview of data collection**

**
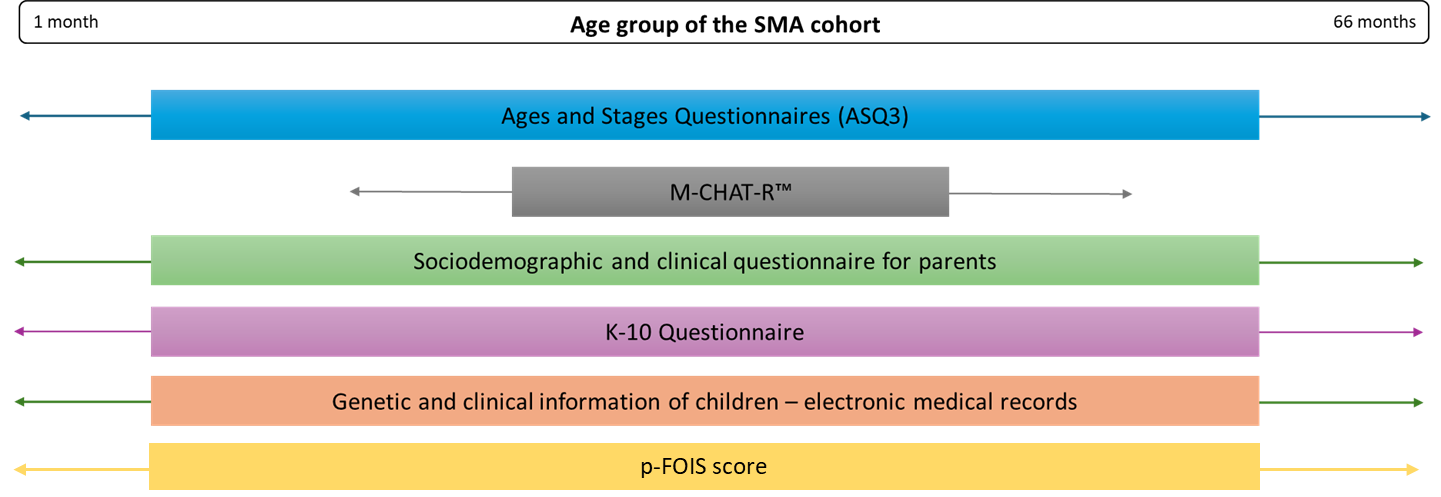
**

*M-CHAT-R: Modified checklist for Autism in Toddlers Revised; K-10: Kessler 10; p-FOIS: Paediatric Functional Oral Intake Scale (also known as CEDAS: Children’s Eating and Drinking Activity Scale)

**Supplementary Table 2. Heatmap of ASQ-3* scores in children with SMA based on diagnostic modality, clinical status and *SMN2* copy number**

| ***Participant number*** | ***Developmental domain*** | | | | |
| --- | --- | --- | --- | --- | --- |
|  | ***Communication*** | ***Gross motor*** | ***Fine motor*** | ***Problem solving*** | ***Personal social*** |
| **Clinical referral with symptoms** | | | | | |
| 1 |  |  |  |  |  |
| 2 |  |  |  |  |  |
| 3 |  |  |  |  |  |
| 4 |  |  |  |  |  |
| 5 |  |  |  |  |  |
| 6 |  |  |  |  |  |
| **Diagnosis through NBS, *2SMN2* and symptomatic** | | | | | |
| 7 |  |  |  |  |  |
| 8 |  |  |  |  |  |
| 9 |  |  |  |  |  |
| 10 |  |  |  |  |  |
| **Diagnosis through NBS, *2SMN2* and clinically silent** | | | | | |
| 11 |  |  |  |  |  |
| 12 |  |  |  |  |  |
| 13 |  |  |  |  |  |
| 14 |  |  |  |  |  |
| 15 |  |  |  |  |  |
| 16 |  |  |  |  |  |
| 17 |  |  |  |  |  |
| 18 |  |  |  |  |  |
| 19 |  |  |  |  |  |
| 20 |  |  |  |  |  |
| 21 |  |  |  |  |  |
| 22 |  |  |  |  |  |
| **Diagnosis through NBS, 3*SMN2* and clinically silent** | | | | | |
| 23 |  |  |  |  |  |
| 24 |  |  |  |  |  |
| 25 |  |  |  |  |  |
| 26 |  |  |  |  |  |
| 27 |  |  |  |  |  |
| 28 |  |  |  |  |  |
| 29 |  |  |  |  |  |
| 30 |  |  |  |  |  |
| 31 |  |  |  |  |  |
| 32 |  |  |  |  |  |
| 33 |  |  |  |  |  |
| 34 |  |  |  |  |  |
| 35 |  |  |  |  |  |
| 36 |  |  |  |  |  |
| 37 |  |  |  |  |  |

*ASQ-3 Ages and Stages Questionnaire 3^rd^ edition

White = ASQ score indicative of on-schedule development (≥1 SD below age-matched population mean); Gray = ASQ score indicative of low developmental risk (1 to 2 SD below age-matched population mean); Black = ASQ score indicative of risk of delay (≤2SD below age-matched population mean)

**Supplementary Table 3. Heatmap of ASQ-3* scores in children with SMA based on bulbar function**

| ***Participant number*** | ***Developmental domain*** | | | | |
| --- | --- | --- | --- | --- | --- |
|  | ***Communication*** | ***Gross motor*** | ***Fine motor*** | ***Problem solving*** | ***Personal social*** |
| **p-FOIS score of 1** | | | | | |
| 1 |  |  |  |  |  |
| **p-FOIS score of 2** | | | | | |
| 2 |  |  |  |  |  |
| 7 |  |  |  |  |  |
| **p-FOIS score of 4.5** | | | | | |
| 4 |  |  |  |  |  |
| 12 |  |  |  |  |  |
| 14 |  |  |  |  |  |
| **p-FOIS score of 5** | | | | | |
| 16 |  |  |  |  |  |
| 10 |  |  |  |  |  |
| 3 |  |  |  |  |  |
| 6 |  |  |  |  |  |
| **p-FOIS score of 6** | | | | | |
| 5 |  |  |  |  |  |
| 8 |  |  |  |  |  |
| 9 |  |  |  |  |  |
| 11 |  |  |  |  |  |
| 13 |  |  |  |  |  |
| 15 |  |  |  |  |  |
| 17 |  |  |  |  |  |
| 18 |  |  |  |  |  |
| 19 |  |  |  |  |  |
| 20 |  |  |  |  |  |
| 21 |  |  |  |  |  |
| 22 |  |  |  |  |  |
| 23 |  |  |  |  |  |
| 24 |  |  |  |  |  |
| 25 |  |  |  |  |  |
| 26 |  |  |  |  |  |
| 27 |  |  |  |  |  |
| 28 |  |  |  |  |  |
| 29 |  |  |  |  |  |
| 30 |  |  |  |  |  |
| 31 |  |  |  |  |  |
| 32 |  |  |  |  |  |
| 33 |  |  |  |  |  |
| 34 |  |  |  |  |  |
| 35 |  |  |  |  |  |
| 36 |  |  |  |  |  |
| 37 |  |  |  |  |  |

*ASQ-3 Ages and Stages Questionnaire 3^rd^ edition

White = ASQ score indicative of on-schedule development (≥1 SD below age-matched population mean); Gray = ASQ score indicative of low developmental risk (1 to 2 SD below age-matched population mean); Black = ASQ score indicative of risk of delay (≤2SD below age-matched population mean)

**Supplementary File 1. Full R code used for the regression analyses**

Contents:
- Adaptive LASSO regression using glmnet
- Bootstrap function for variable selection stability
- Dummy coding for categorical predictors
- Ridge regression for adaptive weights

Instructions:
- This script is written in R and requires the following packages: glmnet, dplyr, tidyr.
- To reproduce the results:
 1. Load your dataset into an object named dat.
 2. Ensure the relevant variables are in columns 3–15.
 3. Run the script sequentially.

## Adaptive LASSO

# Adaptive lasso example https://ricardocarvalho.ca/post/lasso/

# Prepare data set
# Predictor matrix x hold all candidate predictor variables
# The set of predictors variables in the dataset were in columns 3-15
x<- dat[,3:15]
# Dummy-coding modality
x<- x %>%
 mutate(modality2=ifelse(modality==2,1,0)) %>%
 mutate(modality3=ifelse(modality==3,1,0)) %>%
 mutate(modality4=ifelse(modality==4,1,0)) %>%
 dplyr::select(!modality)
x <- model.matrix(~.-1, data= x)
asq <- as.matrix(dat$asq2)
y <- factor(asq)

## Ridge Regression to create the Adaptive Weights Vector
set.seed(999)
cv.ridge <- cv.glmnet(x, y, family='binomial', alpha=0, parallel=TRUE, standardize=TRUE)
w3 <- 1/abs(matrix(coef(cv.ridge, s=cv.ridge$lambda.min)
 [, 1][2:(ncol(x)+1)] ))^1 ## Using gamma = 1
w3[w3[,1] == Inf] <- 999999999 ## Replacing values estimated as Infinite for 999999999


## Adaptive Lasso
set.seed(2342)
cv.lasso <- cv.glmnet(x, y, family='binomial', alpha=1, parallel=TRUE, standardize=TRUE,
 nfolds=37,type.measure='deviance', penalty.factor=w3)

coef(cv.lasso, s=cv.lasso$lambda.1se)
coef <- coef(cv.lasso, s='lambda.1se')
selected_attributes <- (coef@i[-1]+1)


####### Bootstrap function

bootstrap_varsel<-function(){

 for (i in 1:1000) {


 dat_forsample <- dat %>%
 mutate(select=row_number())
 select<-sample(1:37)
 select<- as.data.frame(select)
 boot.samp <- select %>%
 left_join(dat_forsample, by="select")%>%
 dplyr::select(!select)

 #modality is factor with 4 categories. make dummy vars.
 x<- boot.samp[,3:15]
 x<- x %>%
 mutate(modality2=ifelse(modality==2,1,0)) %>%
 mutate(modality3=ifelse(modality==3,1,0)) %>%
 mutate(modality4=ifelse(modality==4,1,0)) %>%
 dplyr::select(!modality)
 x <- model.matrix(~.-1, data= x)
 asq <- as.matrix(boot.samp$asq2)
 y <- factor(asq)

 ## Ridge Regression to create the Adaptive Weights Vector
 #set.seed(999)
 cv.ridge <- cv.glmnet(x, y, family='binomial', alpha=0, parallel=TRUE, standardize=TRUE)
 w3 <- 1/abs(matrix(coef(cv.ridge, s=cv.ridge$lambda.min)
 [, 1][2:(ncol(x)+1)] ))^1 ## Using gamma = 1
 w3[w3[,1] == Inf] <- 999999999 ## Replacing values estimated as Infinite for 999999999

 ## Adaptive Lasso
 #set.seed(999)
 cv.lasso <- cv.glmnet(x, y, family='binomial', alpha=1, parallel=TRUE, standardize=TRUE,
 type.measure='deviance', penalty.factor=w3, nfolds=37)
 coef <- coef(cv.lasso, s='lambda.1se')
 selected_attributes <- (coef@i[-1]+1)

 coeffs <- data.frame(cbind(coef@i, coef@x))
 coeffs_t <- coeffs %>%
 pivot_wider(values_from=X2, names_prefix = "var", names_from = "X1") %>%
 mutate(rep=i)
 # #print(i)
 #coeffs_t
 resulttab<-
 bind_rows(resulttab,coeffs_t)


 }
 return(resulttab)
